# Age-specific differences in influenza virus type and subtype distribution in the 2012/2013 season in 12 European countries

**DOI:** 10.1017/S0950268814003422

**Published:** 2015-02-04

**Authors:** J. BEAUTÉ, P. ZUCS, N. KORSUN, K. BRAGSTAD, V. ENOUF, A. KOSSYVAKIS, A. GRIŠKEVIČIUS, C. M. OLINGER, A. MEIJER, R. GUIOMAR, K. PROSENC, E. STAROŇOVÁ, C. DELGADO, M. BRYTTING, E. BROBERG

**Affiliations:** 1 European Centre for Disease Prevention and Control (ECDC), Solna, Sweden; 2 National Centre of Infectious and Parasitic Diseases, Sofia, Bulgaria; 3 Statens Serum Institut, Copenhagen, Denmark; 4 Institut Pasteur, Paris, France; 5 Hellenic Pasteur Institute, Athens, Greece; 6 Nacionalinė Visuomenės Sveikatos Priežiūros Laboratorija, Vilnius, Lithuania; 7 Laboratoire National de Santé, Dudelange, Luxembourg; 8 National Institute for Public Health and the Environment (RIVM), Bilthoven, The Netherlands; 9 Instituto Nacional de Saúde Doutor Ricardo Jorge, Lisbon, Portugal; 10 National Laboratory for Health, Environment and Food, Laboratory for Public Health Virology, Ljubljana, Slovenia; 11 Public Health Authority of the Slovak Republic, Bratislava, Slovak Republic; 12 Instituto de Salud Carlos III, Madrid, Spain; 13 Public Health Agency of Sweden, Solna, Sweden

**Keywords:** Influenza, influenza (seasonal), respiratory infections, surveillance, surveillance system

## Abstract

The epidemiology of seasonal influenza is influenced by age. During the influenza season, the European Influenza Surveillance Network (EISN) reports weekly virological and syndromic surveillance data [mostly influenza-like illness (ILI)] based on national networks of sentinel primary-care providers. Aggregated numbers by age group are available for ILI, but not linked to the virological data. At the end of the influenza season 2012/2013, all EISN laboratories were invited to submit a subset of their virological data for this season, including information on age. The analysis by age group suggests that the overall distribution of circulating (sub)types may mask substantial differences between age groups. Thus, in cases aged 5–14 years, 75% tested positive for influenza B virus whereas all other age groups had an even distribution of influenza A and B viruses. This means that the intepretation of syndromic surveillance data without age group-specific virological data may be misleading. Surveillance at the European level would benefit from the reporting of age-specific influenza data.

## INTRODUCTION

Influenza is an acute respiratory tract infection caused by influenza viruses. The most commonly involved genera are influenza viruses A and B [1]. Influenza A viruses are further divided in subtypes – mainly A(H1)pdm09 and A(H3) in recent influenza seasons – and influenza B viruses are classified in lineages. In the Northern hemisphere, these viruses are responsible for the yearly influenza epidemic that infects a substantial proportion (~20%) of the population during winter [2]. Although most cases of infection will be asymptomatic or suffer a relatively mild illness, the large numbers of infected inviduals result in large numbers of severe cases and deaths every season [1, 3].

The epidemiology of seasonal influenza is influenced by age on different levels. First, the highest notification rates are usually reported in younger age groups [4, 5]. Second, the clinical presentation of the disease varies across age groups [1, 2]. The typical sudden onset of fever accompanied by headache, malaise, myalgia and upper respiratory symptoms seen in adults and adolescents is not as often observed in children or elderly people [1]. Third, adults aged ⩾65 years are more at risk for complications, hospitalizations and death [6, 7]. Fourth, there is some evidence that some age groups play a more prominent role in transmitting the virus. It has been suggested that children may play an important role in household transmission [8], that they may even be a driver for the spread of influenza epidemics, although the magnitude of children's contribution remains controversial [9]. Finally, immunity against influenza is likely to differ across age groups because of different previous exposure to influenza viruses as highlighted during the 2009 pandemic when elderly people were less affected [10], different vaccination policies [11] and different vaccine effectiveness [12]. Seasonal vaccination is recommended for risk groups, including the elderly, in most European Union/European Economic Area (EU/EEA) countries, but children are included only in a minority of Member States [11]. Low vaccine effectiveness has been reported in recent influenza seasons, especially in older age groups [12].

Circulating influenza strains influence the age distribution of infection, disease and severe outcomes of each season. A recent study carried out in the UK showed that the risk of symptomatic disease tends to decrease with age for influenza A and children have higher rates during influenza B waves [2]. The A(H1)pdm09 subtype appears to have infected children first with adolescents and young adults being significantly affected only in the second wave of the 2009 pandemic [2]. Some influenza subtypes, such as influenza A(H3N2), could be associated with a higher severity, especially in older age groups [13, 14] and in long-term healthcare facilities [15]. Influenza types and subtypes may have an impact on the age distribution of hospitalized cases [16, 17], but this is not well documented in primary care.

Influenza surveillance in Europe, with the integration of clinical and virological data as its cornerstone, has a long history [18]. Virological and syndromic surveillance data [mostly influenza-like illness (ILI) but also acute respiratory illness (ARI)] are collected through national networks of sentinel primary-care providers. Aggregated numbers by age group are available for ILI/ARI, but not for the virological data.

The objectives of this study were to (*a*) describe age-specific differences in the distribution of influenza viruses for the influenza season 2012/2013, and (*b*) compare age distributions of influenza-positive sentinel cases and ILI/ARI cases.

### METHODS

The surveillance of influenza in Europe is performed by the European Influenza Surveillance Network (EISN) under the coordination of the European Centre for Disease Prevention and Control (ECDC) [16].

At the end of the influenza season 2012/2013, all EISN laboratories were invited to submit a subset of their data for this season. The variables were limited to reporting week, patient age and influenza type and subtype. For the purpose of this analysis, both dual influenza infections and infections with influenza virus type C were excluded. ILI and ARI data for the 2012/2013 season were obtained from the European Surveillance System (TESSy) database hosted at ECDC. EISN members are recommended to use the EU case definitions for ILI and ARI [19]. If both ILI and ARI were available, ILI was preferred. The age groups analysed were 0–4, 5–14, 15–64 and ⩾65 years.

For the description of the influenza season 2012/2013, pooled virological data was extracted from TESSy for the 29 EISN countries.

Age distributions of influenza-positive sentinel cases and ILI or ARI notifications were compared at country level. Influenza type and subtype distribution between age groups was compared at European level. In countries with age-specific specimen denominator data, the proportion of influenza-positive specimens was compared across age groups at country level. Overall dominance of virus type and subtype was compared with age-specific dominance at country level. Dominance was defined as a proportion of an influenza virus type or subtype ⩾60%.

Testing of differences was done with *χ*^2^ or Fisher's exact tests with the level of significance set at *P* < 0·05.

## RESULTS

### Participating countries and available data

Twelve of 29 EISN Member States reported age-specific data for 7890 positive specimens during the reporting period from week 40/2012 to week 20/2013. Most participating countries reported ILI cases, but France and Bulgaria only reported ARI (Table 1). In nine countries, ILI/ARI data were available by age group (Table 1). Nine countries were able to provide denominators with the number of tests performed (Table 2). Twenty-one co-infections and two cases with a positive test for influenza virus type C were excluded. Information on age was not available for 18 cases from six countries. Finally, 7849 cases (99%) were kept in the analysis.

**Table 1.** Age distribution of influenza-positive sentinel specimens and ILI/ARI cases by country, 12 European Union countries, influenza season 2012/2013

Reporting country	Type of surveillance	Proportion of specimens and cases by age group, total number of specimens and cases					Comparison between age distributions in confirmed influenza and ILI/ARI cases (*P* values)
		0–4 yr (%)	5–14 yr (%)	15–64 yr (%)	⩾65 yr (%)	*N*	
Bulgaria	Specimens	18	44	35	3	79	<0·01
	ARI cases	21	24	50	5	99 565	
Denmark	Specimens	6	13	75	6	171	<0·01
	ILI cases	13	18	59	10	7358	
France	Specimens	26	27	42	5	2602	<0·01
	ARI cases	34	24	35	7	162 536	
Greece	Specimens	11	44	36	9	88	n.a.*
	ILI cases						
Lithuania	Specimens	10	27	54	9	367	n.a.
	ILI cases						
Luxembourg	Specimens	16	30	50	4	353	n.a.
	ILI cases						
The Netherlands	Specimens	5	16	70	9	387	<0·01
	ILI cases	13	10	59	18	2149	
Portugal	Specimens	4	13	76	7	193	0·67
	ILI cases	5	10	76	8	221	
Slovakia	Specimens	9	32	56	2	133	<0·05
	ILI cases	18	28	48	6	225 559	
Slovenia	Specimens	15	38	45	2	338	<0·01
	ILI cases	7	36	55	2	541	
Spain	Specimens	12	32	53	4	2651	<0·01
	ILI cases	17	34	45	5	17 741	
Sweden	Specimens	5	14	74	7	487	0·62
	ILI cases	5	13	73	9	1302	

ILI, Influenza-like illness; ARI, acute respiratory illness.

* Comparisons were not applicable (n.a.) where syndromic cases were not reported by age group.

**Table 2.** Age distribution of the proportion of influenza-positive specimens by country, nine European Union countries with age-specific specimen denominator data, influenza season 2012/2013

Reporting country	Proportion of influenza-positive specimens/tested specimens by age group					Comparison between positivity rates (*P* values)
	0–4 yr (%)	5–14 yr (%)	15–64 yr (%)	⩾65 yr (%)	Total (%)	
Bulgaria	14/94 (15)	35/141 (25)	28/164 (17)	2/21 (10)	79/420 (19)	0·15
France	668/1767 (38)	700/1140 (67)	1095/2203 (50)	139/298 (47)	2602/5 408 (48)	<0·01
Greece	10/33 (30)	39/85 (46)	32/67 (48)	7/16 (50)	88/201 (44)	0·39
The Netherlands	21/130 (16)	60/122 (49)	271/776 (35)	35/148 (24)	387/1176 (33)	<0·01
Portugal	7/14 (50)	26/45 (58)	149/205 (73)	11/61 (18)	193/325 (59)	<0·01
Slovakia	12/19 (63)	43/72 (60)	75/106 (71)	3/7 (43)	133/204 (66)	0·25
Slovenia	49/158 (31)	128/194 (66)	153/242 (63)	8/11 (73)	338/605 (56)	<0·01
Spain	317/773 (41)	836/1352 (62)	1401/2562 (55)	97/256 (38)	2651/4943 (54)	<0·01
Sweden	26/101 (26)	66/121 (55)	360/1217 (30)	35/209 (17)	487/1648 (30)	<0·01

### Influenza season 2012/2013

**Fig. 1.**
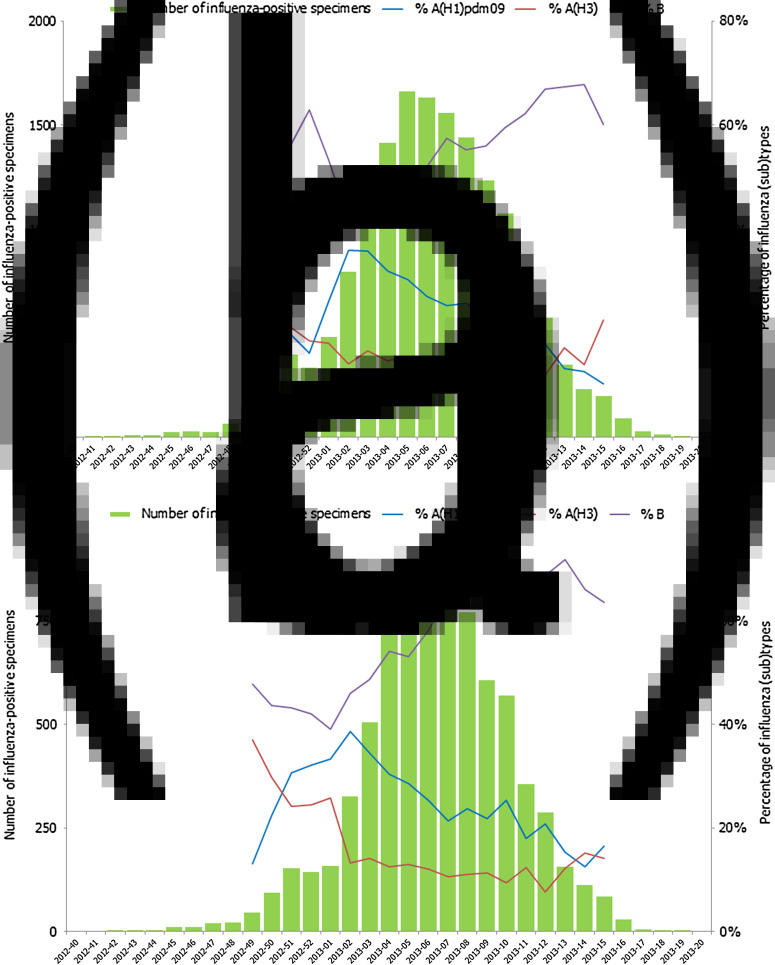
Number of influenza-positive sentinel specimens and percentage* by type, (sub)type and week. (*a*) European Union/European Economic Area (EU/EEA), (*b*) 12 EU countries†, week 40/2012 to week 20/2013. (* Percentages are displayed in weeks where at least 50 influenza-positive specimens were reported; † Bulgaria, Denmark, France, Greece, Lithuania, Luxembourg, The Netherlands, Portugal, Slovakia, Slovenia, Spain, and Sweden.

At the EU/EEA level, the 2012/2013 influenza season started about week 49/2012, had a prolonged peak between week 4/2013 and week 8/2013 and lasted until week 16/2013 (Fig. 1*a*). The epidemic peak occurred in week 6/2013, when 1632 of the 2607 sentinel swabs collected (63%) tested positive for influenza virus in 29 countries. From week 40/2012 to week 20/2013, these 29 countries tested 33 819 specimens, of which 15 744 (47%) tested positive for influenza virus.

Similarly, in the 12 participating countries, the 2012/2013 influenza season started about week 49/2012, had a prolonged peak between week 4/2013 and week 8/2013 and lasted until week 16/2013 (Fig. 1*b*). In week 7/2013, at the peak of the epidemic, 892 of the 1336 sentinel swabs collected (67%) tested positive for influenza virus. From week 40/2012 to week 20/2013, the 12 participating countries tested 16 508 specimens, of which 8076 (49%) tested positive for influenza virus.

In the 12 participating countries as well as in all EU/EEA countries, the distribution of influenza virus (sub)types over the season showed an overall predominance of influenza B virus with an increase over time (~70% of influenza-positive specimens were B virus for week 13/2013). Influenza A(H1)pdm09 virus peaked in week 2/2013 when it accounted for 40% of all influenza-positive specimens while the proportion of A(H3) virus was near or below 20% for most of the season (Fig. 1*a*, *b*).

### Influenza detections and clinical diagnoses by age group

Of the nine countries for which ILI/ARI data were available by age group, seven had an age distribution based on influenza-positive sentinel specimens that was statistically different from the one observed for ILI/ARI (Table 1). With the exception of Slovenia, the proportion of influenza-positive specimens in the ⩾65 years age group was systematically lower than the proportion of ILI/ARI.

The proportion of tested specimens by age group was statistically different from the one observed for ILI/ARI in all eight countries for which both number of tested specimens and ILI/ARI data were available by age group. However, in all these eight countries the 15–64 years age group accounted for both the highest number of ILI/ARI and tested specimens.

The proportion of influenza-positive specimens significantly differed accross age groups in six of the nine countries that provided age-specific specimen denominator data (Table 2). In some countries, substantial differences were observed as for The Netherlands, where 16% of specimens were positive in the 0–4 years group compared to 49% in the 5–14 years group.

### Distribution of influenza virus type and subtype by age group

**Fig. 2.**
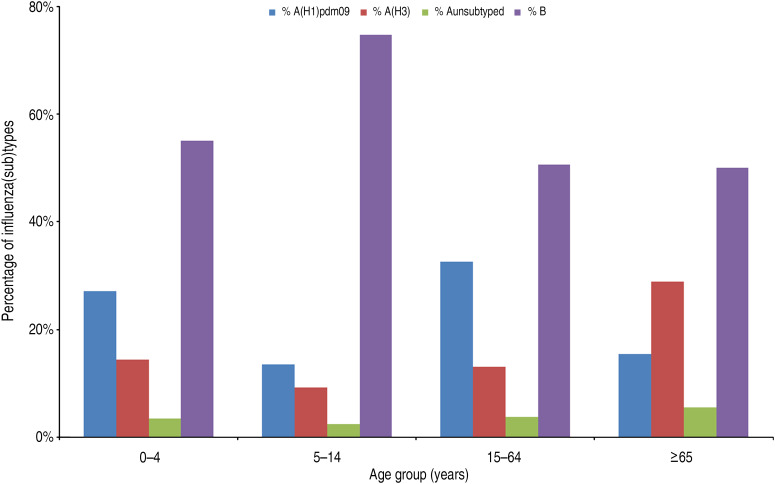
Influenza virus type and subtype distribution by age group, 12 European Union countries, influenza season 2012/2013.

Of the 7849 positive specimens reported with information on age, 1227 (16%) were from the 0–4 years age group, 2161 (28%) from 5–14 years, 4067 (52%) from 15–64 years and 394 (5%) from cases aged ⩾65 years. In cases aged 5–14 years, 75% tested positive for influenza B virus whereas all other age groups had an even distribution of influenza A and B viruses (Fig. 2). Of the influenza A viruses subtyped, A(H1)pdm09 viruses dominated over A(H3) viruses in all cases up to age 64 years (68% *vs*. 32% overall) whereas in those aged ⩾65 years, A(H3) viruses dominated (65% *vs*. 35%). Overall, the distribution of the virus types and subtypes were significantly different in age groups (*P* < 0·0001).

In most countries, dominant virus (sub)type varied accross age groups (Tables 3 and 4). Of note, a predominance of influenza B virus was observed in most countries (10/12) in the 5–14 years age group while no clear pattern emerged in the other age groups. Spain was the only country where predominance of the same type was observed in all age groups.

**Table 3.** Age distribution of influenza-positive specimens by influenza virus (sub)type and country, 12 European Union countries, influenza season 2012/2013

Reporting country	Virus (sub)type	0–4 yr (%)	5–14 yr (%)	15–64 yr (%)	⩾65 yr (%)	Total (%)
Bulgaria	A(H1)pdm09	6 (43)	5 (14)	8 (29)	1 (50)	20 (25)
	A(H3)	0	3 (9)	3 (11)	0	6 (8)
	A unsubtyped	0	2 (6)	0	0	2 (3)
	B	8 (57)	25 (71)	17 (61)	1 (50)	51 (65)
	Total	14 (100)	35 (100)	28 (100)	2 (100)	79 (100)
Denmark	A(H1)pdm09	1 (10)	0	11 (9)	0	12 (7)
	A(H3)	6 (60)	5 (23)	47 (36)	5 (50)	63 (37)
	A unsubtyped	0	0	0	0	0
	B	3 (30)	17 (77)	71 (55)	5 (50)	96 (56)
	Total	10 (100)	22 (100)	129 (100)	10 (100)	171 (100)
France	A(H1)pdm09	173 (26)	95 (14)	322 (29)	17 (12)	607 (23)
	A(H3)	122 (18)	99 (14)	225 (21)	56 (40)	502 (19)
	A unsubtyped	14 (2)	9 (1)	45 (4)	4 (3)	72 (3)
	B	359 (54)	497 (71)	503 (46)	62 (45)	1421 (55)
	Total	668 (100)	700 (100)	1095 (100)	139 (100)	2602 (100)
Greece	A(H1)pdm09	4 (40)	8 (21)	16 (50)	1 (14)	29 (33)
	A(H3)	6 (60)	28 (72)	13 (41)	6 (86)	53 (60)
	A unsubtyped	0	0	0	0	0
	B	0	3 (8)	3 (9)	0	6 (7)
	Total	10 (100)	39 (100)	32 (100)	7 (100)	88 (100)
Lithuania	A(H1)pdm09	23 (61)	31 (31)	99 (50)	6 (18)	159 (43)
	A(H3)	5 (13)	11 (11)	5 (3)	3 (9)	24 (7)
	A unsubtyped	7 (18)	14 (14)	49 (25)	5 (15)	75 (20)
	B	3 (8)	43 (43)	44 (22)	19 (58)	109 (30)
	Total	38 (100)	99 (100)	197 (100)	33 (100)	367 (100)
Luxembourg	A(H1)pdm09	21 (38)	26 (24)	83 (47)	1 (7)	131 (37)
	A(H3)	1 (2)	1 (1)	6 (3)	1 (7)	9 (3)
	A unsubtyped	12 (22)	14 (13)	27 (15)	9 (64)	62 (18)
	B	21 (38)	66 (62)	61 (34)	3 (21)	151 (43)
	Total	55 (100)	107 (100)	177 (100)	14 (100)	353 (100)
The Netherlands	A(H1)pdm09	6 (29)	4 (7)	78 (29	8 (23)	96 (25)
	A(H3)	11 (52)	12 (20)	54 (20)	16 (46)	93 (24)
	A unsubtyped	0	0	0	0	0
	B	4 (19)	44 (73)	139 (51)	11 (31)	198 (51)
	Total	21 (100)	60 (100)	271 (100)	35 (100)	387 (100)
Portugal	A(H1)pdm09	1 (14)	6 (23)	87 (58)	7 (64)	101 (52)
	A(H3)	1 (14)	1 (4)	4 (3)	1 (9)	7 (4)
	A unsubtyped	0	0	0	0	0
	B	5 (71)	19 (73)	58 (39)	3 (27)	85 (44)
	Total	7 (100)	26 (100)	149 (100)	11 (100)	193 (100)
Slovakia	A(H1)pdm09	6 (50)	5 (12)	18 (24)	0	29 (22)
	A(H3)	1 (8)	5 (12)	13 (17)	1 (33)	20 (15)
	A unsubtyped	2 (17)	2 (5)	7 (9)	0	11 (8)
	B	3 (25)	31 (72)	37 (49)	2 (67)	73 (55)
	Total	12 (100)	43 (100)	75 (100)	3 (100)	133 (100)
Slovenia	A(H1)pdm09	22 (45)	24 (19)	82 (54)	5 (63)	133 (39)
	A(H3)	3 (6)	6 (5)	12 (8)	0	21 (6)
	A unsubtyped	0	3 (2)	0	0	3 (1)
	B	24 (49)	95 (74)	59 (39)	3 (38)	181 (54)
	Total	49 (100)	128 (100)	153 (100)	8 (100)	338 (100)
Spain	A(H1)pdm09	58 (18)	78 (9)	371 (26)	11 (11)	518 (20)
	A(H3)	10 (3)	17 (2)	58 (4)	12 (12)	97 (4)
	A unsubtyped	6 (2)	8 (1)	11 (1)	2 (2)	27 (1)
	B	243 (77)	733 (88)	96 169	7274	2009 (76)
	Total	317 (100)	836 (100)	1 401 100	97 100	2 651 100
Sweden	A(H1)pdm09	11 (42)	11 (17)	151 (42)	3 (9)	176 (36)
	A(H3)	11 (42)	12 (18)	89 (25)	13 (38)	125 (26)
	A unsubtyped	1 (4)	0	15 (4)	2 (6)	18 (4)
	B	3 (12)	43 (65)	106 (29)	16 (47)	168 (34)
	Total	26 (100)	66 (100)	361 (100)	34 (100)	487 (100)
Total	A(H1)pdm09	332 (27)	293 (14)	1326 (33)	60 (15)	2011 (26)
	A(H3)	177 (14)	200 (9)	529 (13)	114 (29)	1020 (13)
	A unsubtyped	42 (3)	52 (2)	154 (4)	22 (6)	270 (3)
	B	676 (55)	1616 (75	2059 (51)	197 (50)	4548 (58)
	Total	1227 (100)	2161 (100)	4068 (100)	393 (100)	7849 (100)

**Table 4.** Dominant influenza virus type or subtype by age group and country, 12 European Union countries, influenza season 2012/2013

Reporting country	Overall dominant* type or subtype	Dominant type or subtype by age group			
		0–4 yr	5–14 yr	15–64 yr	⩾65 yr
Bulgaria	B	None	B	B	None
Denmark	None	A(H3)	B	None	None
France	None	None	B	None	None
Greece	A(H3)	A(H3)	A(H3)	None	A(H3)
Lithuania	None	A(H1)pdm09	None	None	None
Luxembourg	None	None	B	None	None
Netherlands	None	None	B	None	None
Portugal	None	B	B	None	A(H1)pdm09
Slovakia	None	None	B	None	B
Slovenia	None	None	B	None	A(H1)pdm09
Spain	B	B	B	B	B
Sweden	None	None	B	None	None

* Dominance was defined as ⩾60%.

## DISCUSSION

In Europe, the influenza season 2012/2013 was characterized by a co-circulation of three influenza virus types and subtypes, offering a good opportunity to determine whether the distribution of types and subtypes differs accross age groups [20].

Our findings suggest that the overall distribution of types and subtypes may mask substantial differences between age groups. Thus, A(H3) – a subtype frequently associated with a higher morbidity than the previous seasonal A(H1) and B viruses [13, 14] – was especially prevalent in cases aged ⩾65 years. Conversely, influenza B virus predominated in children aged 5–14 years, which supports prevous findings from Germany and the UK [2, 21]. The remaining age groups experienced a more evenly distributed mixed season of A and B viruses. Of note, country-specific profiles of dominance and age-specific proportions of influenza virus types and subtypes were markedly different.

Age-specific influenza surveillance data should be interpreted with caution. In most countries, the age distribution of ILI/ARI differed from the age distribution of cases with confirmed influenza. Our findings suggest that sampling strategies may have slighly differed accross age groups. The relative overrepresentation of positive specimens collected in adults aged 15–64 years may be suggestive of differences in health-seeking behaviour. Since both clinical and virological indicators are collected through the same sentinel scheme, there are at least two additional possible explanations. First, the contribution of other respiratory pathogens differs across age groups. Besides influenza A and B viruses, parainfluenza virus and respiratory syncytial virus (RSV) contribute to ILI estimates [22]. Specifically, the burden of RSV in children aged <5 years is now well established [23] and there is increasing evidence suggesting that it might also cause a substantial amount of illness in the elderly [24]. Second, the varying proportion of positive specimens across age group could be explained by differential virus shedding by age or virus (sub)type. A recent study carried out in Germany could not find any evidence supporting this latter hypothesis [25]. Regardless of the reason why age distributions differs between clinical and confirmed cases, this study reminds us that ILI/ARI rates by age group should be interpreted very cautiously in the absence of virological data. In countries where ILI/ARI data are not available by age group, influenza surveillance would therefore benefit from collecting virological data by age group.

If confirmed over several influenza seasons, these findings may have important implications for vaccination policies. The first objective of influenza vaccination is to prevent severe disease and death. It is now well established that elderly people have the highest mortality risk and therefore constitute a well-identified risk group [26]. Previous studies have suggested that the extension of vaccine coverage to younger age groups would increase protection in the elderly [27, 28]. In seasons with different dominating strains accross age groups, the impact of such an extension may well be limited.

The relatively low number of specimens collected in some countries was an important limitation of this study, as some statistical analyses were not possible. For the same reason, it was not possible to look at the dynamics of transmission across age groups. In addition, such analysis would probably have to include several seasons. This study also underlined the need to document the sampling strategies in EISN countries as the selection of patients to be swabbed may not be systematic in all surveillance schemes.

The overall distribution of influenza viruses in the 29 EISN Member States under surveillance was similar to the one observed in the 12 countries participating in this study, suggesting that our findings may also be valid for the rest of the EISN Member States.

## CONCLUSION

This study demonstrates the added value of including age group data in routine virological influenza surveillance because they provide a better indication of the age-specific distribution of influenza infection and its causative virus types and subtypes than pooled virological data or age-specific ILI/ARI rates. With better estimates of the burden of influenza in different age groups, it would help identify target groups for preventive measures. Last, similar data collection in the coming years could help improve our understanding of the dynamics and transmission of influenza. Such data would not necessary have to be collected every week, but could be collected once after every season. This would also constitute a step towards a more integrated clinical and virological influenza surveillance.
